# TBC1D1 represses glioma progression by altering the integrity of the cytoskeleton

**DOI:** 10.18632/aging.205377

**Published:** 2024-01-05

**Authors:** Jiahong Cai, Yong’an Jiang, Peng Chen, Jiawei Liang, Yi Zhang, Raorao Yuan, Hengyi Fan, Yuefei Zhong, Jianhui Cai, Shiqi Cheng, Yan Zhang

**Affiliations:** 1 Department of Neurosurgery, The Second Affiliated Hospital of Nanchang University, Nanchang 330000, Jiangxi, China; 2 Department of Neurology, Shang Rao GuangXin District People’s Hospital, Shangrao 334100, Jiangxi, China; 3 Department of Neurosurgery, Nanchang County People’s Hospital, Nanchang 330200, Jiangxi, China

**Keywords:** TBC1D1, glioma, invasion, migration, cytoskeleton

## Abstract

Background: Glioma is one of the most aggressive malignant brain tumors and is characterized by invasive growth and poor prognosis. TBC1D1, a member of the TBC family, is associated with the development of various malignancies. However, the role of TBC1D1 in glioma-genesis remains unclear.

Methods: The effect of TBC1D1 on the prognosis of glioma patients and related influencing factors were analyzed in the Chinese Glioma Genome Atlas (CGGA) and The Cancer Genome Atlas (TCGA) databases. Expression of TBC1D1 in glioma cell lines was detected by western blotting. Cell viability and proliferation were measured by EdU and Colony formation assays, respectively. Transwell and wound healing assays were performed to determine the cell migration and invasion capacities. Immunofluorescence was used to observe actin morphology in the cytoskeleton.

Results: We discovered that high TBC1D1 expression in gliomas led to poor prognosis. Downregulation of TBC1D1 in glioma cells significantly inhibited multiple important functions, such as proliferation, migration, and invasion. We further demonstrated that the tumor-inhibitory effect of TBC1D1 might occur through the P-LIMK/cofilin pathway, destroying the cytoskeletal structure and affecting the depolymerization of F-actin, thereby inhibiting glioma migration.

Conclusion: TBC1D1 affects the balance and integrity of the actin cytoskeleton via cofilin, thereby altering the morphology and aggressiveness of glioma cells. This study provides a new perspective on its role in tumorigenesis, thereby identifying a potential therapeutic target for the treatment of gliomas.

## INTRODUCTION

Glioma is the most common primary intracranial malignant tumor, accounting for approximately 41–44.6% of all intracranial tumors [1]. Glioblastoma has the highest degree of malignancy, and has an extremely poor prognosis. It has a median overall survival (OS) of less than 14 months, and a 5-year survival rate less than 5% [2, 3]. Standard treatment includes maximum tumor resection, in addition to adjuvant radiation, and chemotherapy. Tumors are aggressive and invasive; therefore, the total surgical resection rate is low. In addition, the residual tumor tissue is resistant to radiotherapy and chemotherapy [4], and traditional treatment is limited. Therefore, in recent years, molecular-targeted therapy has become popular in glioma research.

The degree of malignancy in gliomas is related to the cytoskeleton [5, 6]. This is a network of fibers in eukaryotic cells involved in cell shape maintenance and cell motility. Cell shape is dictated by the cytoskeleton, comprising microtubules, microfilaments, and intermediate filaments. Significantly, microfilaments, primarily constituted by f-actin (fibrous actin), form a central component of the cytoskeleton [7]. F-actin, in particular, plays a crucial role in influencing the migration of glioma cells [8]. Filopodia are aggregated by f-actin, which is closely related to tumor invasiveness [6]. Re-organization of the actin cytoskeleton is associated with tumor cell migration and the morphological changes of tumor cells [9–12]. Therefore, research focusing on the signaling pathways that cause changes in the cytoskeleton and related proteins, involved in the development of gliomas can direct research into glioma-targeted therapy.

TBC1D1 is a member of TBC family. It has been reported that TBC1D3 is amplified in prostate cancer [13, 14]. TBC1D8 amplification drives the occurrence of ovarian cancer [15], and TBC1D15 is upregulated in liver, prostate, and thyroid cancers [16]. However, there are no studies on the correlation between TBC1D1 expression and glioma prognosis. This study first predicted the relationship between TBC1D1 and the prognosis of glioma using the CGGA (http://www.cgga.org.cn/help.jsp) [17] and TCGA (https://portal.gdc.cancer.gov/) [18] databases, then studied the effect of TBC1D1 downregulation on the proliferation, migration, and invasion of glioma cells. In addition, it further analyzed whether these phenomena are related to changes in the glioma cytoskeleton.

## MATERIALS AND METHODS

### Bioinformatic analysis

All patients with glioma were collected from the CGGA and TCGA. Gene Expression Profiling Interactive Analysis (GEPIA, http://gepia.cancer-pku.cn) contains expression data from tumor and non-tumor samples [19]. We then verified TBC1D1 expression in glioma cell lines by western blotting.

### Cell culture and transfection

The glioma cell lines T98G and LN229 were purchased from Shanghai Hanwei Biotechnology Co., Ltd. (Shanghai, China). The cell lines were cultured in DMEM medium supplemented with 10% fetal bovine serum (FBS), 100 units/mL penicillin, and 100 μg/mL streptomycin. The culture conditions were: 37°C with 5% CO_2_. Transfection of siRNA (silencing RNA, siRNA) (General Biol Co., Ltd., Anhui, China) was performed using Lipofectamine 3000 (Invitrogen, Carlsbad, CA, USA). The siRNA sequences of TBC1D1 are listed in Table 1. Transfection procedures were performed according to the manufacturer’s instructions. Fetal bovine serum (FBS), DMEM, and antibiotics were purchased from Gibco (Grand Island, NY, USA).

**Table 1:** siRNA sequences of TCB1D1.

**Gene**	**Sequence (5′–3′)**
siRNA1 Top strand	GGCUAUUCUUCAACAGAUATT
siRNA1 Bottom strand	UAUCUGUUGAAGAAUAGCCTT
siRNA2 Top strand	CUCAUUAGCGGACACAAUATT
siRNA2 Bottom strand	UAUUGUGUCCGCUAAUGAGTT
siRNA3 Top strand	CGAGGUUGCUUCAUGAUUATT
siRNA3 Bottom strand	UAAUCAUGAAGCAACCUCGTT
siRNA-NC Top strand	UUCUCCGAACGUGUCACGUTT
siRNA-NC Bottom strand	ACGUGACACGUUCGGAGAATT

### Cell proliferation assay

EdU assay was performed using the YF^®^594 Click-iT EdU Kit (UElandy, Suzhou, China) at 24 h after the transfected 1 × 10^4^ cells were plated into 96-well plates. After EdU labeling, the percentage of EdU-positive cells was visualized under a fluorescence microscope (Nikon ECLIPSE Ti2-U), as described previously [20].

### Colony formation analysis

Transfected cells were seeded into 6-well plates (500 cells/well) and cultured in a complete culture medium for 2 weeks. When approximately 50 cell clusters were observed under a microscope, the 6-well plates were removed from the incubator. Cell colonies were washed with PBS, fixed in 4% paraformaldehyde for 30 min, and stained with crystal violet for 15 min, at room temperature. Subsequently, the colonies were washed with ultra-pure water, dried upside down, and photographed.

### Wound-healing

To assess the migratory and invasive abilities of glioma cells *in vitro*, wound-healing assays were performed. For the wound-healing assay, glioma cells were planted in 6-well cell culture plates. A scratching step was vertically performed on the center of each well, using 200 μl pipettes. The cells were cultured for 24 h, 48 h in DMEM without FBS. Images were captured using a microscope at 100×. The gap distance was measured by plotting on the software scale. The proportion of change was calculated and analyzed using statistics.

### Transwell for cell migration and invasion

For the transwell assay, cells were cultured in transwell chambers with 8 μm pores in 24-well plates. For the migration assay, 2000 transfected cells were suspended in 200 μL serum-free medium and added to the upper transwell chamber. After incubation for 12 h in a humidified atmosphere containing 5% CO_2,_ at 37°C, the migrated cells that had adhered to the lower surface of the membrane were fixed in 4% paraformaldehyde for 30 min. Thereafter, they were stained with crystal violet for 15 min at room temperature. The number of migrating cells was counted in five randomly selected fields at 200× magnification using a microscope. For the invasion assay, transwell chambers were coated with Matrigel (Corning Co., Ltd., Wujiang, China), following the same procedures as those for the migration assay [20].

### Immunofluorescence and cytoskeleton co-staining

Glioma cells were seeded in 24-well plates and fixed in 4% formaldehyde diluted in 1× PBS for 15 min. The cells were then washed thrice with 1× PBS for 5 min. Samples were blocked in 5% BSA for 60 mins and incubated with the primary antibody overnight, at 4°C. After washing with PBS, the secondary antibody was added and incubated at room temperature for 2 h in the dark. Images were captured and analyzed using a fluorescence microscope (Nikon ECLIPSE Ti2-U).

### Western blot

Total protein was extracted using RIPA lysis buffer (1:1000) (Solarbio Science and Technology Co., Ltd., Beijing, China) and protease/phosphate inhibitors (Solarbio Science and Technology Co., Ltd., Beijing, China). The protein concentrations were measured using a bicinchoninic acid protein quantification kit (Solarbio Science and Technology Co., Ltd., Beijing, China). Proteins were separated by 10% or 12.5% SDS-PAGE gel electrophoresis, transferred to PVDF membranes, and probed with primary antibodies TBC1D1 (diluted at 1:1000, Proteintech, China), N-Cadherin, E-Cadherin (diluted at 1:1000, CST, USA), MMP-2, MMP-9 (diluted at 1:1000), Profilin (diluted at 1:10000), Cofilin, Cofilin (phosphoS3) (diluted at 1:1000, Abcam, UK), and Phospho-LIMK1/LIMK2 (Thr508, Thr505) (diluted at 1:1000, Invitrogen). The membranes were probed with horseradish peroxidase-conjugated secondary antibodies. An enhanced chemiluminescence detection system (Bio-Rad, Hercules, CA, USA) was used for protein detection. Anti-GAPDH antibody and anti-β-TUBULIN (diluted at 1:2000, Proteintech) were used to monitor the loading amount.

### Statistics analysis

GraphPad Prism software was used for data analysis. ImageJ was used for figure analysis. *T*-test was used for comparing the statistical data of the two groups. All data are displayed as mean ± standard deviation (SD). The cell experiments were performed at least three times. ^*^P value < 0.05 was considered to be statistically significant.

## RESULTS

### Factors related to TBC1D1 on survival in glioma patients

**Figure 1:**
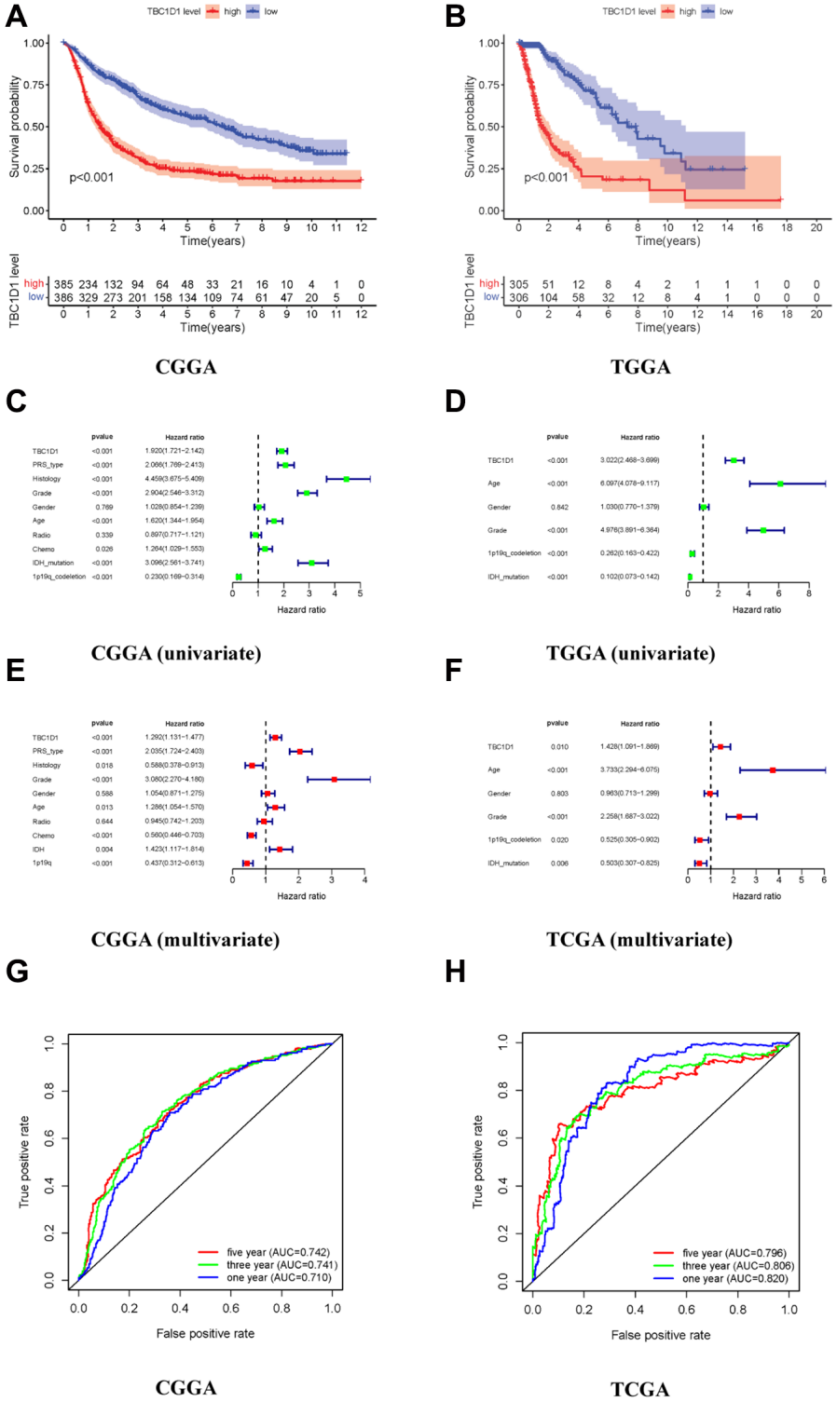
**High expression of TBC1D1 in gliomas leads to poor prognosis.** (**A**, **B**) Relationship between TBC1D1 expression and prognosis in CGGA and TCGA databases. (**C**, **D**) Multivariate analysis in CGGA and TCGA databases. (**E**, **F**) Multivariate analysis in CGGA and TCGA databases. (**G**, **H**) ROC curves in CGGA and TCGA databases, diagnostic value of TBC1D1 expression in gliomas.

To explore the impact of TBC1D1 on the prognosis of individuals with glioma, we conducted an analysis using patient data retrieved from the CGGA and TCGA databases. The outcomes derived from both databases indicated a notable disparity in survival rates between patients exhibiting high TBC1D1 expression and those with low expression, with a significantly lower survival rate observed in the former group (Figure 1A, 1B). We performed univariate and multivariate analyses using the COX regression model to analyze clinical characteristics and TBC1D1 expression data. Univariate analysis showed that the prognosis of patients with glioma in the CGGA database was associated with the expression of TBC1D1, PRS type, histology, grade, age, and IDH mutations (Figure 1C). It was related to high TBC1D1 expression, age, grade, and IDH mutation in the TCGA database (Figure 1D). Subsequently, a multivariate analysis employing a COX regression model was undertaken. The findings, as observed in the CGGA database, revealed an association between the prognosis of glioma patients and several factors, including high TBC1D1 expression, PRS type, and grade (Figure 1E). In the TCGA database, it was related to TBC1D1 high expression, age, and grade (Figure 1F). The results showed that high TBC1D1 expression may be an important factor in the poor prognosis of gliomas.

### Clinical diagnostic value of TBC1D1

The ROC curve was established to verify the expression of TBC1D1 in glioma tissues and the results of the database analysis. The three-year and five-year curved survival regions (AUCs) of patients with high expression in TBC1D1 were 0.741 and 0.742 in the CGGA database, respectively (Figure 1G). The AUC under the three-year and five-year curves in the TCGA database for high TBC1D1 expression were 0.806 and 0.796, respectively, (Figure 1H). This suggests that the expression level of TBC1D1 has credible value in predicting the prognosis of gliomas.

### Correlation between TBC1D1 expression and clinical characteristics in glioma patients

**Figure 2:**
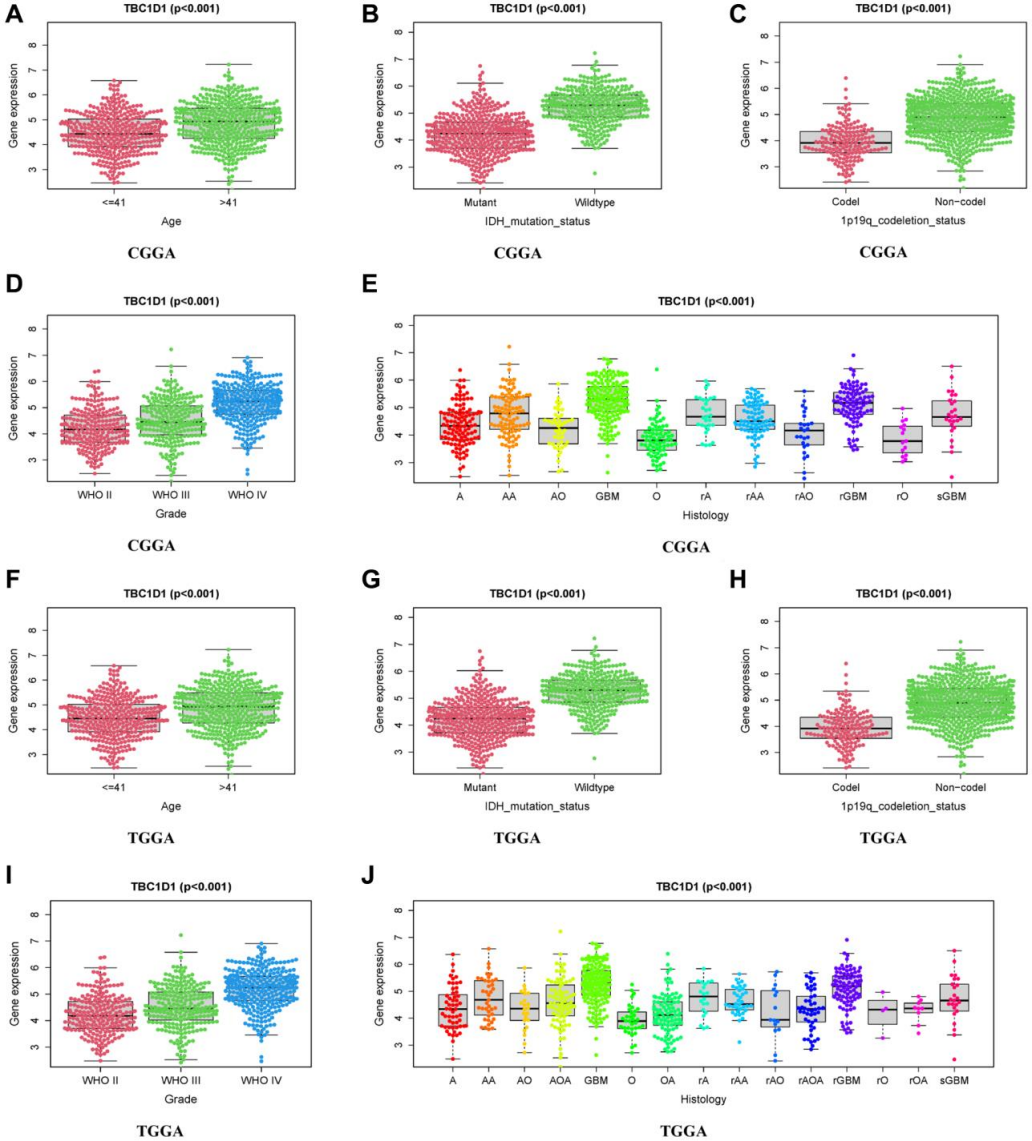
**Relationship between TBC1D1 expression and clinical features.** (**A**–**E**) Relationship of TBC1D1 expression to age, IDH mutation status, 1p19q codeletion status, grade, histology in CGGA databases. (**F**–**J**) Relationship between TBC1D1 expression and age, IDH mutation status, 1p19q co-deletion status, grade, and histology in the TCGA database.

Several clinical correlation analyses related to TBC1D1 were performed using the CGGA and TCGA datasets. In the CGGA and TCGA databases, TBC1D1 expression levels were significantly correlated with age, grade, histology, IDH mutation status, and 1p19q co-deletion status (Figure 2A–2J).

### Expression of TBC1D1 in gliomas

**Figure 3:**
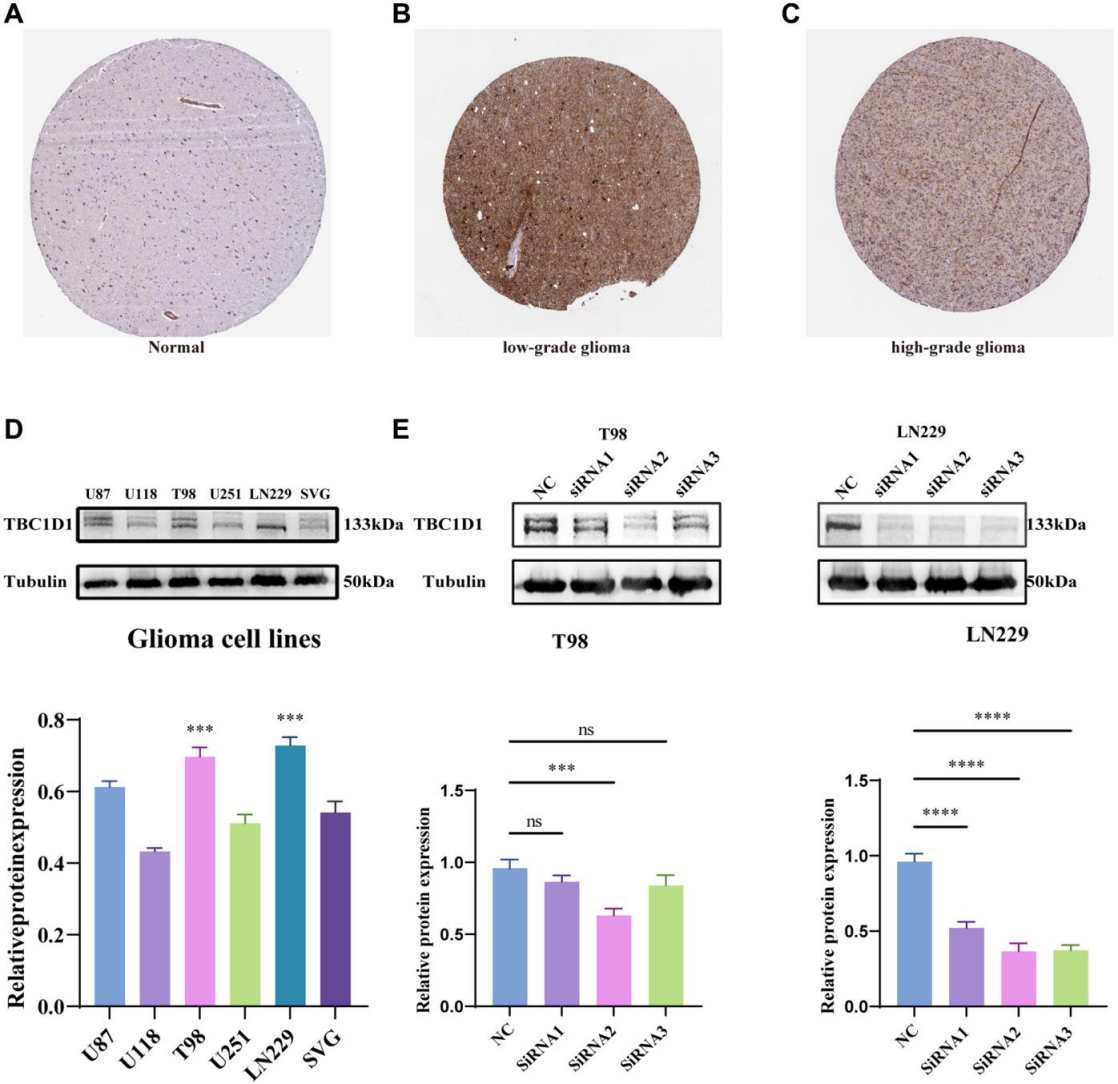
**Immunohistochemistry results obtained from the HPA project database, expression of TBC1D1 across tissues and expression of TBC1D1 in glioma cell lines.** (**A**) Protein expression of TBC1D1 in normal brain tissues. (**B**) Expression of TBC1D1 in low-grade glioma tissues. (**C**) Expression of TBC1D1 in high-grade glioma tissues. (**D**) Protein expression of TBC1D1 in glioma cell lines. (**E**) Knockdown of TBC1D1 by siRNA in T98 and LN229. (All data were presented as mean ± s.d. from three independent experiments (^*^P < 0.05, ^**^P < 0.01, ^***^P < 0.005, ^****^P < 0.001).

Immunohistochemical data from the HPA project (https://www.proteinatlas.org/) suggested that TBC1D1 protein levels were increased in low-grade and high-grade glioma tissues compared with those in normal cerebral cortex tissues (Figure 3A–3C). The expression of TBC1D1 proteins was also increased in the SVG and glioma cell lines (U87, U118, T98G, U251, and LN229), especially in T98G and LN229 cells (Figure 3D). Consequently, T98G and LN229 cells were chosen for *in vitro* experiments to validate our findings. In order to delineate the impact of TBC1D1 on the malignant behavior of glioma cells, we executed siRNA-mediated knockdown of TBC1D1 in both T98G and LN229 cell lines. SiRNA1, siRNA2, and siRNA3 were used and siRNA2 was selected for subsequent experiments (Figure 3E).

### TBC1D1 knockdown inhibited the proliferation of glioma cells

**Figure 4:**
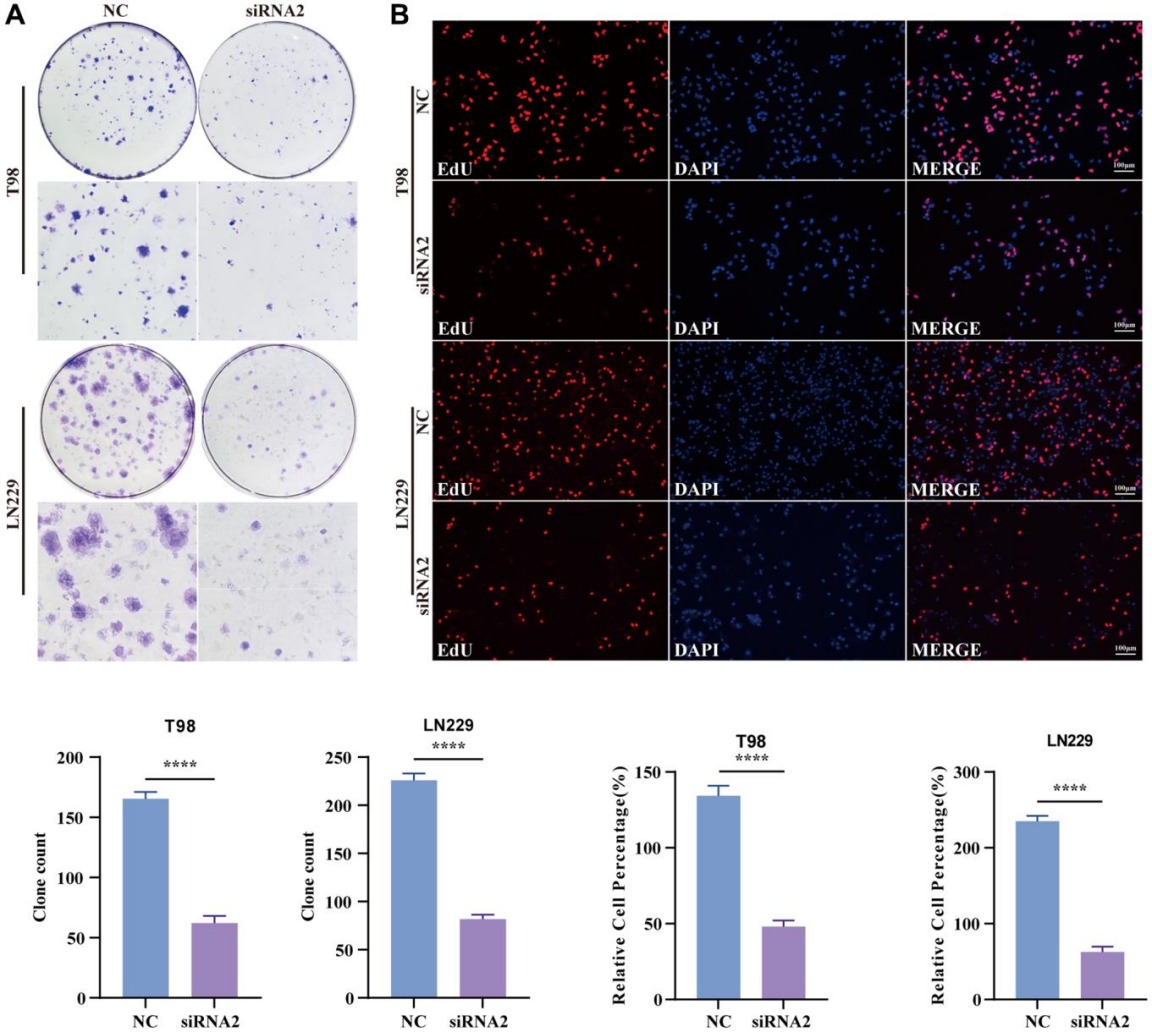
**Knockdown TBC1D1 inhibits the proliferation of glioma cells.** (**A**) Detection of proliferation of transfected cells by colony formation experiments. (**B**) Detection of proliferation of transfected cells by EdU staining (Scale bar = 100 μm). (All data were presented as mean ± s.d. from three independent experiments (^*^P < 0.05, ^**^P < 0.01, ^***^P < 0.005, ^****^P < 0.001).

A colony formation assay was conducted to assess the potential impact of TBC1D1 on glioma cell proliferation. The findings indicated a substantial inhibition of cell growth in both T98G and LN229 cells upon the downregulation of TBC1D1 expression (Figure 4A). To confirm that the reduction in cell proliferation resulted from the downregulation of TBC1D1, an EdU cell proliferation assay was performed. Upon downregulation of TBC1D1 expression in both cell lines, a notable reduction in the count of EdU-positive cells was observed (Figure 4B).

### TBC1D1 knockdown suppressed the migration and invasion of glioma cells

**Figure 5:**
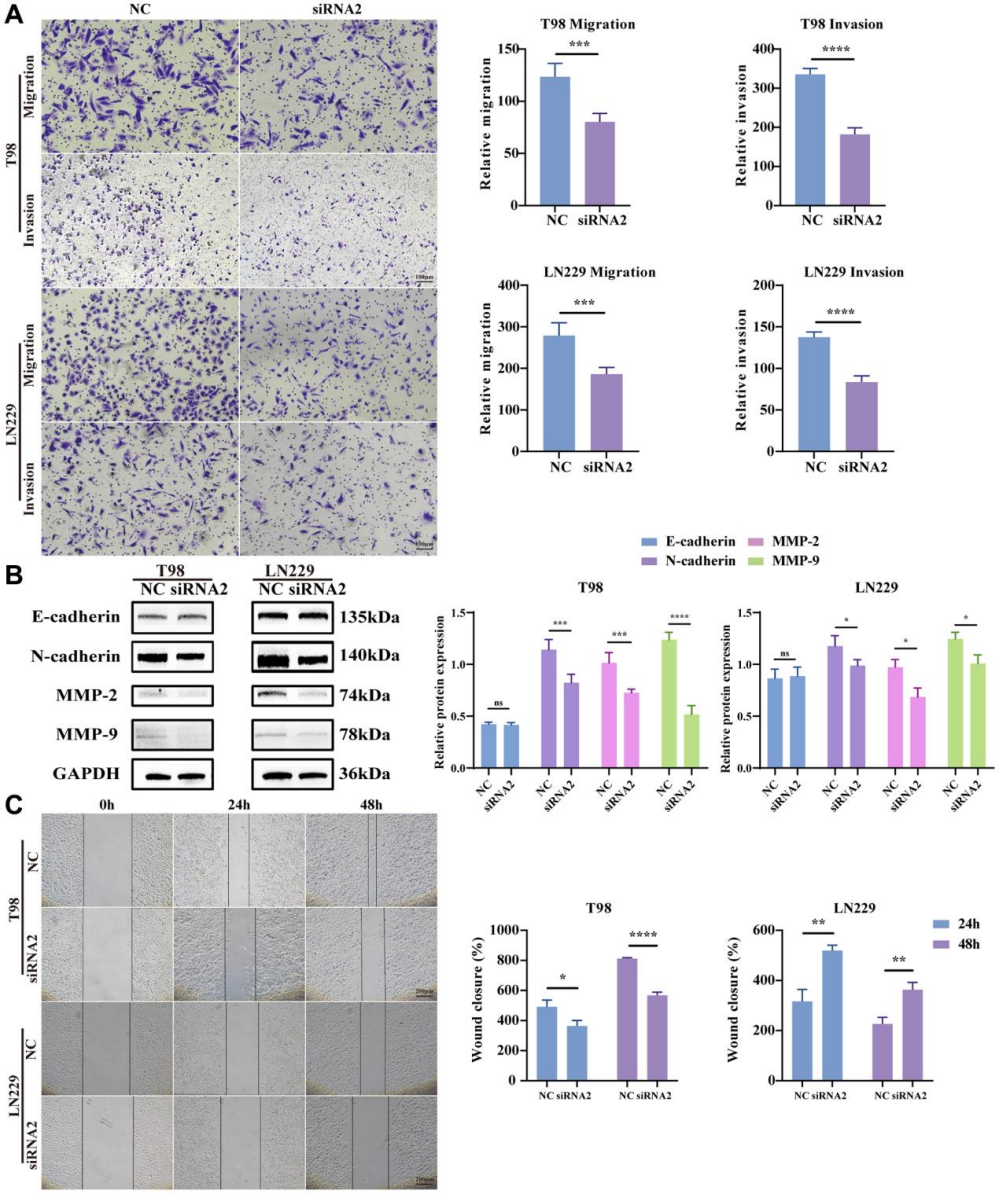
**Knockdown TBC1D1 inhibits glioma cell migration and invasion.** (**A**) Detection of migration and invasion of transfected cells by transwell method (Scale bar = 100 μm). (**B**) Detection of E-cadherin, N-cadherin, MMP-2 and MMP-9 protein expression. (**C**) Perform wound-healing assay to detect migration of transfected cells (Scale bar = 200 μm). (All data were presented as mean ± s.d. from three independent experiments (^*^P < 0.05, ^**^P < 0.01, ^***^P < 0.005, ^****^P < 0.001).

Being recognized as one of the most formidable malignant brain tumors, glioma is distinguished by its highly infiltrative growth, extending extensively into surrounding tissues without defined boundaries [21]. Hence, to scrutinize the potential involvement of TBC1D1 in the migration and invasion of glioma cells, transwell and wound healing assays were conducted. The outcomes revealed a decrease in the number of migrating cells with TBC1D1 downregulation compared to the control cells. Furthermore, the count of invading cells demonstrated a reduction with downregulated TBC1D1 compared to the control group (Figure 5A). There was no significant change in the expression of E-cadherin, however, the expression of N-cadherin, MMP-2, and MMP-9 decreased, further indicating that the downregulation of TBC1D1 affected the MMP family and thus weakened the aggressiveness of gliomas (Figure 5B). Moreover, the healing of scratch wounds was notably impeded following the knockdown of TBC1D1 in both cell lines during 24 hours and 48 hours of incubation (Figure 5C).

### Effects of TBC1D1 knockdown on the cytoskeleton

**Figure 6:**
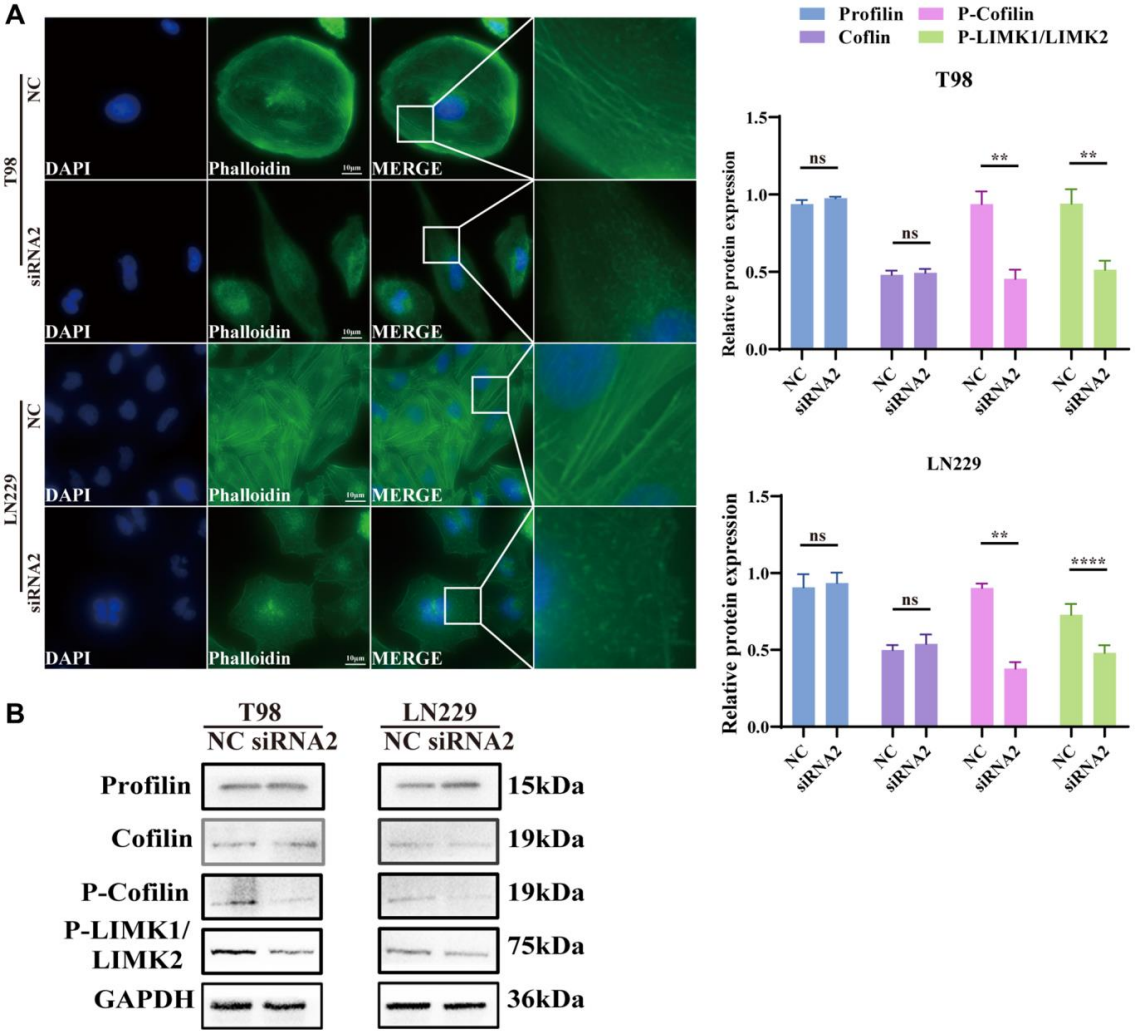
**Knockdown TBC1D1 affects the integrity of the F-actin.** (**A**) Fluorescence microscopy was used to observe the morphological changes of F-actin cytoskeleton stained with phalloidin after transfection (Scale bar = 10 μm). (**B**) Detection of profilin, cofilin, P-LIMK1Thr508/P-LIMK2Thr505 and P-cofilinSer3 protein expression. (All data were presented as mean ± s.d. from three independent experiments (^*^P < 0.05, ^**^P < 0.01, ^***^P < 0.005, ^****^P < 0.001).

The cytoskeleton, vital for sustaining cell morphology and facilitating migration, prompted our hypothesis that TBC1D1 influences cell migration through its impact on cytoskeletal proteins. Staining F-actin with phalloidin-iFluor 488 reagent and subsequent observation under a fluorescence microscope revealed that the microfilaments in untreated tumor cells displayed intact filaments. In contrast, those in the TBC1D1 downregulated group exhibited signs of fragmentation and aggregation (Figure 6A).

Profilin is a conserved actin-binding protein that regulates the rate of actin polymerization by binding to actin monomers and promoting the exchange of adenosine diphosphate (ADP) with ATP [22]. Cofilin induces depolymerization at the negative end of microfilaments, hindering their reassembly. Nevertheless, self-inhibition ensues following phosphorylation at the Ser3 site [23]. LIM kinases, specifically LIMK1 and LIMK2, represent serine/threonine kinases characterized by the presence of two zinc-finger motifs within their N-terminal regulatory domains [22]. Activated LIMK phosphorylates cofilin at the Ser3 residue, thereby inhibiting its depolymerization activity [24]. The effects of profilin and cofilin were not obvious, but P-LIMK1Thr508/P-LIMK2Thr505 and P-cofilin^Ser3^ decreased (Figure 6B). We further performed immunofluorescence analysis of cofilin and P-cofilin^Ser3^. P-cofilin^Ser3^ was decreased in the cytosol but was not significantly altered in the nucleus. The cofilin content did not change significantly in either the nucleus or the cytosol (Supplementary Figure 1). The findings suggest that the diminution of TBC1D1 disrupts the cytoskeleton of glioma cells through the P-LIMK/cofilin pathway, consequently influencing the migratory behavior of tumor cells.

## DISCUSSION

Gliomas, prevalent malignancies originating in the central nervous system, are distinguished by their rapid progression, unfavorable prognostic outlook, and a proclivity for recurrent occurrences. Due to its robust capacity to infiltrate neighboring tissues, achieving a positive prognosis for patients proves challenging [25]. Gaining deeper insights into the mechanisms underlying the occurrence and development of gliomas is of paramount importance. This requires exploring novel pathogenic mechanisms and protein markers, identifying fresh targets, and comprehensively enhancing the postoperative quality of life for patients with glioblastoma. Recent findings indicate that both lncRNA ARST and E3 Ubiquitin Ligase RNF139 possess inhibitory effects on glioma progression [26]. TBC family proteins exhibit close associations with the onset and progression of diverse types of tumors. Most of these proteins function as oncoproteins and participate in the regulation of malignant transformation or metastasis of cells. The involvement of TBC/Rab-GAPs in malignant tumors is likely intricately connected to the modulation of Rabs. These proteins play a crucial role in facilitating the transport and circulation of receptor proteins both intercellularly and between cells and the extracellular matrix, consequently influencing the process of cell invasion. Additionally, many components of cancer gene signaling pathways, such as EGFR, RAS, and RAC, are localized to endocytic vesicles or adhesion sites [27, 28]. This study marks the initial exploration of the impact of TBC1D1 on the progression of gliomas, with preliminary investigations. *In vitro* phenotype experiments were conducted to validate this prediction. In this investigation, we initially examined the CGGA and TCGA databases. The analysis revealed that elevated TBC1D1 expression in glioma is associated with an unfavorable prognosis and holds clinical diagnostic significance in predicting glioma outcomes. We proceeded to investigate if heightened TBC1D1 expression independently serves as a risk factor impacting glioma prognosis. Through both univariate and multivariate analyses, we identified factors relevant to the adverse prognosis of glioma. This further proved the value of this gene in the diagnosis of glioma.

In investigating this hypothesis, we attenuated the expression of TBC1D1 within glioma cell lines. Our observations revealed that the attenuated expression of TBC1D1 exerted a suppressive influence on malignant phenotypic traits, comprising the intricate processes of glioma proliferation, invasion, and migration. The matrix metalloproteinase (MMP) family is involved in the degradation of the extracellular matrix, participating in both normal physiological processes such as embryonic development, reproduction, and tissue remodeling, as well as pathological processes like inflammatory reactions and angiogenesis in tumors [29, 30]. Most MMPs are secreted in the form of inactive protein precursors, which are activated after cleavage by extracellular proteinases. MMP2 and MMP9 belong to the gelatinase subgroup of the matrix metalloproteinase family, serving as type IV collagenases capable of degrading gelatin components in the extracellular matrix, and thereby participating in the metastatic processes of tumors. Numerous studies have demonstrated abnormal expression of MMP2 and MMP9 in tissues following tumor occurrence [31]. Research has also revealed that MMP9 is a crucial regulator of the extracellular matrix and basement membrane, with its expression promoting the differentiation and proliferation of epithelial cells [22, 32]. Moreover, MMP activity has been implicated in cancer progression [33]. The results of this study suggest that the knockdown of TBC1D1 affects the downstream expression of MMP2 and MMP-9 and inhibits the invasiveness of glioma cells. However, how it affects the expression of upstream proteins requires further study.

The cytoskeleton plays a vital role in various cellular activities and F-actin is one of its most important components [34]. Carlier et al. documented the depolymerization of actin filaments facilitated by cofilin, an actin-depolymerizing protein (ADP) [35]. The role of cofilin promotes the fragmentation of aged actin fibers, accelerating the synthesis of new F-actin. Under normal circumstances, the polymerization and depolymerization of F-actin coexist, sustaining a dynamic equilibrium crucial for cellular activities, substance transport, and various other processes. Elevated levels of cofilin intensify the depolymerization and repolymerization of actin fibers, fostering the malignant invasion and migration of gliomas [36]. This, in turn, triggers actin depolymerization upon downregulation of TBC1D1 in glioma cells. Past research has delineated the influence of the P-LIMK/cofilin pathway on the cytoskeleton [22]. Notably, studies have demonstrated that LIM kinases, including LIMK1, LIMK2, TESK1, and TESK2, selectively phosphorylate the N-terminal Ser3 site of cofilin, impeding its interaction with actin [23]. Phosphorylation at the Thr508 and Thr505 sites enhances the kinase activity of LIMK [37]. Conversely, a decrease in the expression of TBC1D1 results in reduced phosphorylation of LIMK, consequently weakening the phosphorylation of cofilin at the Ser3 site [38].

In summary, TBC1D1 exerts influence on the structural integrity of the cytoskeleton via its interaction with cofilin, leading to morphological alterations and changes in the aggressiveness of glioma cells. This investigation introduces a novel perspective on TBC1D1’s involvement in tumorigenesis, highlighting its potential as a therapeutic target for addressing gliomas.

## Supplementary Materials

Supplementary Figure 1
