# Supplementary material for: TBC1D1 represses glioma progression by altering the integrity of the cytoskeleton
Source: Aging (Albany NY). 2024 Jan 5;16(1):431–44. doi: 10.18632/aging.205377 (PMC10817367; doi:10.18632/aging.205377)
Supplement: Supplementary Figure 1 [file aging-16-205377-s001.pdf]

SUPPLEMENTARY FIGURE

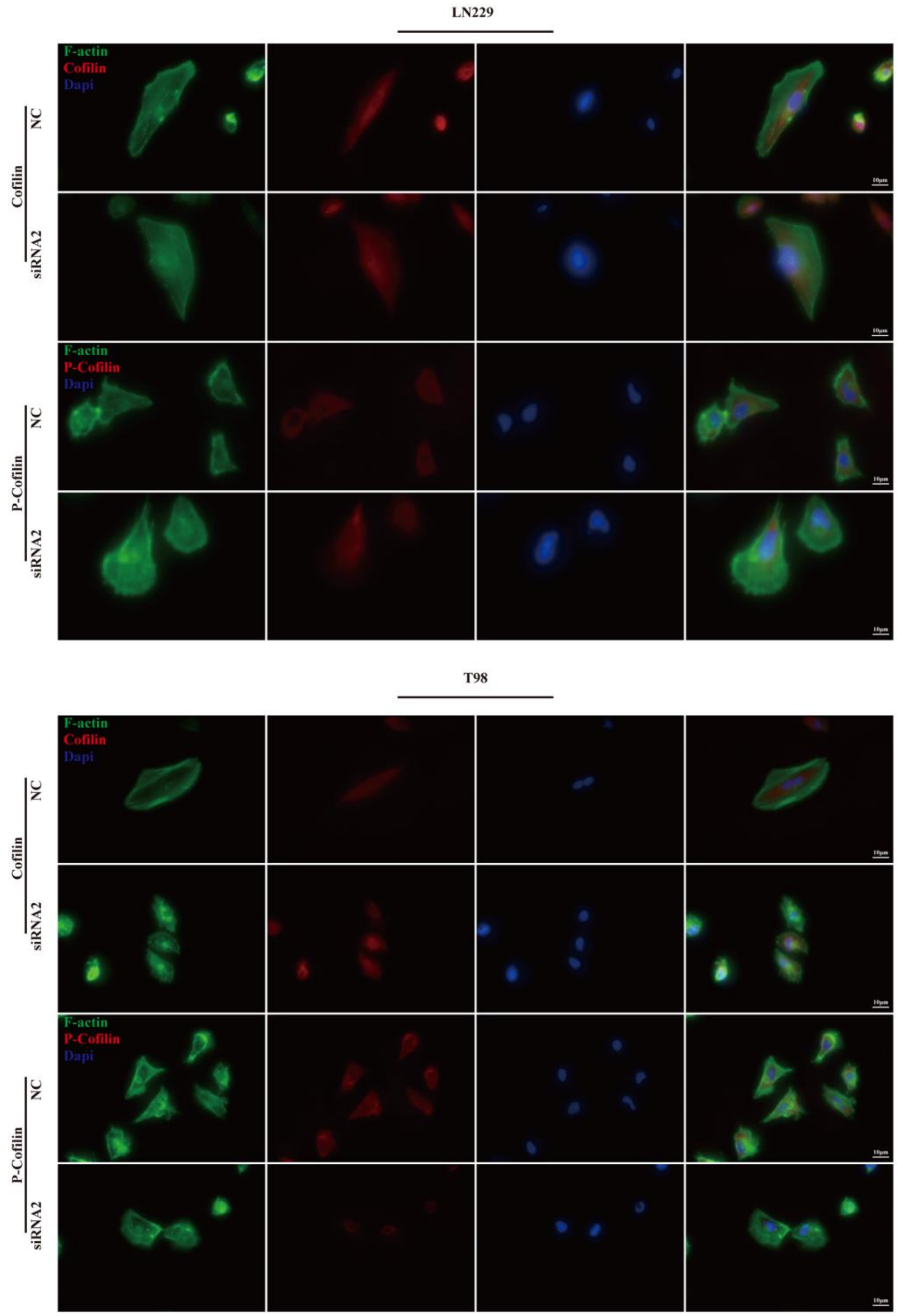

Supplementary Figure 1. Intracellular location of cofilin and p-cofilinSer3 in T98MG and LN229 (Scale bar = 10 μm).
